# Gastric Adenocarcinoma With Enteroblastic Differentiation and Coexisting Well-Differentiated Tubular Adenocarcinoma: A Case Report

**DOI:** 10.7759/cureus.91319

**Published:** 2025-08-30

**Authors:** Kohei Matsuoka, Masaichi Ohira, Hitoshi Teraoka, Haruhito Kinoshita, Taichi Shoji

**Affiliations:** 1 Surgery, Baba Memorial Hospital, Osaka, JPN

**Keywords:** adenocarcinoma with enteroblastic differentiation, collision tumor, gaced, gastric cancer, well-differentiated tubular adenocarcinoma

## Abstract

A 92-year-old male was referred to our hospital owing to progressive anemia and a positive fecal occult blood test. Upper gastrointestinal endoscopy revealed an advanced gastric carcinoma on the posterior wall of the lower gastric body, which was diagnosed as tubular adenocarcinoma by biopsy. The patient underwent an open distal gastrectomy, and the operative specimen showed a 45-mm-sized type 3 lesion on the posterior wall of the lower gastric body and a 35-mm-sized type 0-IIa tumor on the oral side of the type 3 lesion. Histopathological examination revealed that the type 3 lesion contained cells with pale sporangia, and immunohistochemistry was positive for SALL4, α-fetoprotein, and Glypican-3, leading to a diagnosis of gastric adenocarcinoma with enteroblastic differentiation (GACED). The type 0-IIa tumor was negative for these immunostainings and was identified as a well-differentiated tubular adenocarcinoma. There was no mixture of the two tumors except for the border area. Intraoperative peritoneal washing cytology was positive, and the stage was pStage IV. Owing to his advanced age, postoperative chemotherapy was not initiated. At three months postoperatively, lung and liver metastases were detected. We report a case of GACED with coexisting well-differentiated tubular adenocarcinoma.

## Introduction

Gastric adenocarcinoma with enteroblastic differentiation (GACED) is a rare subtype of alpha-fetoprotein (AFP)-producing gastric carcinoma, characterized by tubular, papillary, or solid proliferation of the columnar epithelium with a pale cytoplasm resembling the gastrointestinal epithelium of the early embryonic stage [1]. Owing to its high vascular invasiveness, GACED is associated with a poorer prognosis than conventional gastric adenocarcinoma [2]. GACED often coexists with conventional adenocarcinoma components [3], which can make preoperative diagnosis challenging. Given its aggressive nature, however, early diagnosis and timely therapeutic intervention are crucial. Detailed pathological and clinical reports on cases involving both GACED and conventional adenocarcinoma components remain scarce. Herein, we present a rare case of a collision tumor, in which two distinct tumor types independently arise and come into contact without intermingling, composed of GACED and well-differentiated adenocarcinoma, and discuss its potential histogenesis and clinical implications.

## Case presentation

**Figure 1:**
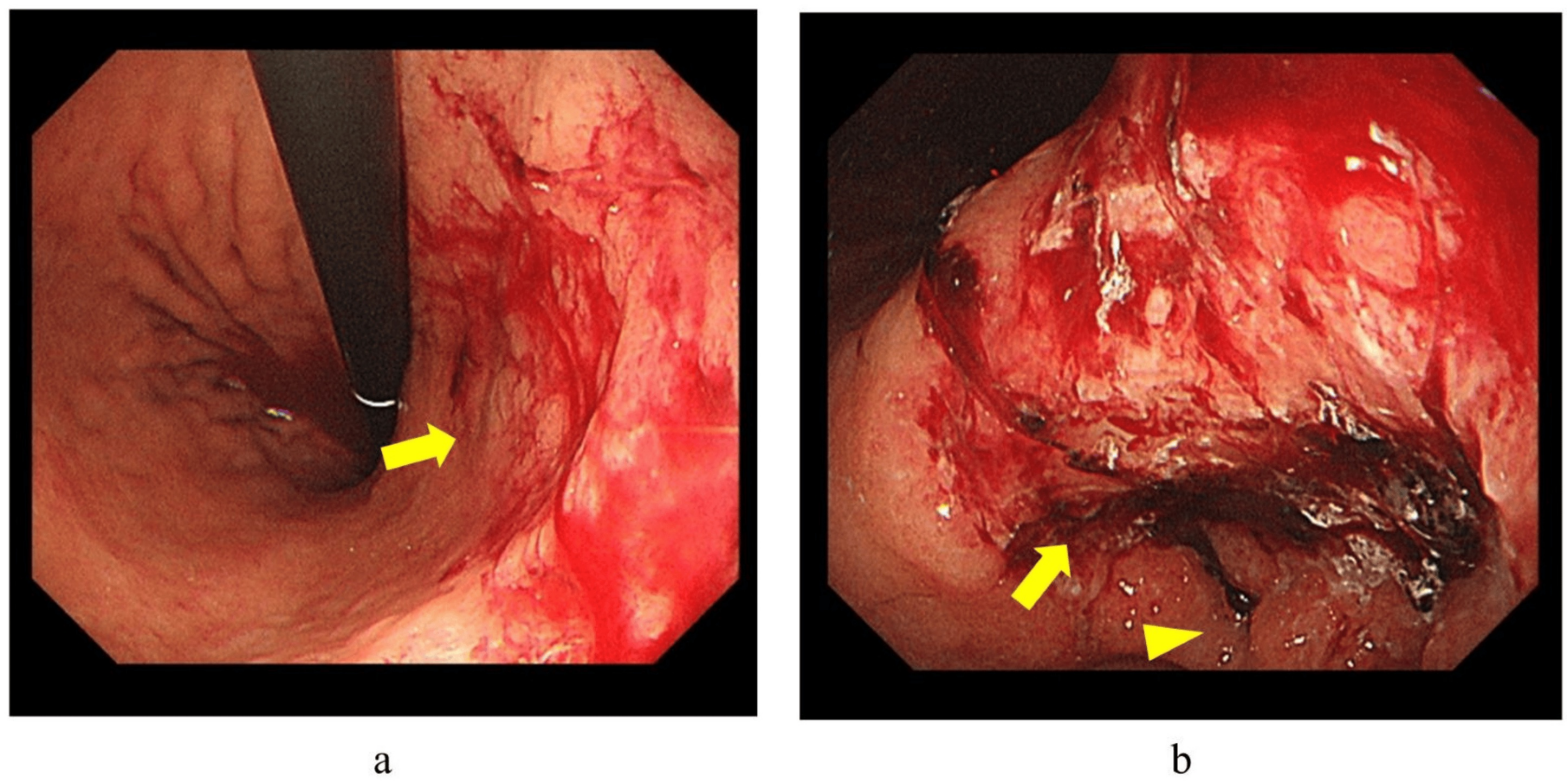
Upper gastrointestinal endoscopy findings (a) Upper gastrointestinal endoscopy showing a type 3 lesion (arrow) located on the posterior wall of the stomach. (b) Type 3 tumor (arrow) extended to the antrum. The pyloric ring is indicated by the arrowhead. No active bleeding was observed.

A 92-year-old man with high blood pressure, dyslipidemia, diabetes, and chronic kidney disease visited our hospital and was closely observed and treated for progressive anemia and positive fecal occult blood test results. At the time of admission, the vital signs were normal. The conjunctiva of the eyelids was pale. The abdomen was flat and soft, without tenderness. Blood biochemical tests revealed anemia (hemoglobin, 7.3 g/dL) and renal dysfunction (blood urea nitrogen, 45.0 mg/dL, creatinine, 2.13 mg/dL). Tumor markers were elevated (carcinoembryonic antigen, 31.2 ng/mL; CA19-9, 88.8 U/mL). Upper gastrointestinal endoscopy revealed type 3 lesions (Bormann classification) extending from the lower stomach to the vestibular region on the posterior wall of the stomach (Figure 1). No active bleeding from the tumor site was observed. After six biopsies at the tumor margin, he was diagnosed with well-differentiated tubular adenocarcinoma.

**Figure 2:**
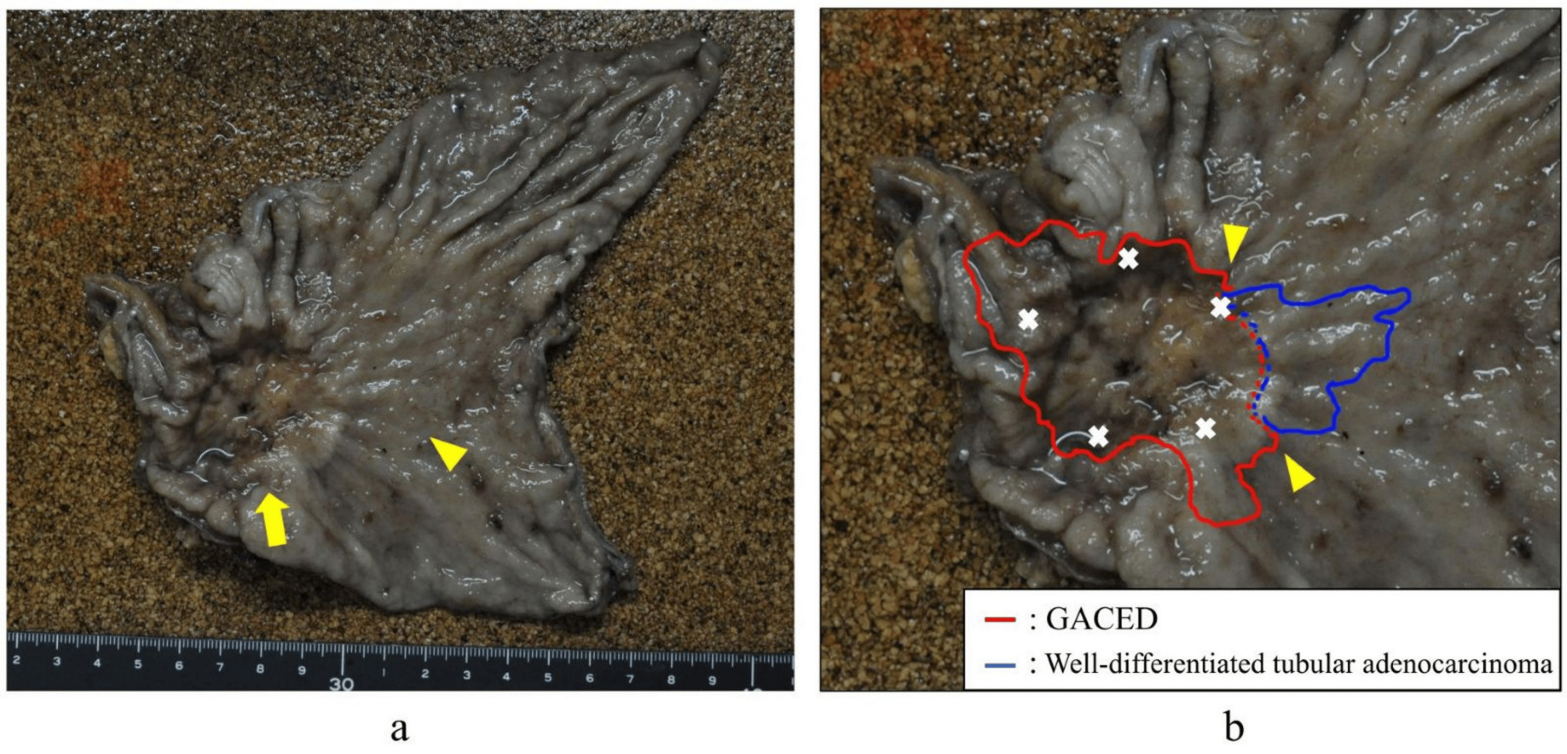
Findings of the surgical specimen (a) A 45 × 45-mm-sized type 3 lesion (arrow) is observed in the lower gastric body, and a 35-mm-sized type 0–IIa lesion (arrowhead) extends contiguously to the oral side of the type 3 lesion. (b) The gastric adenocarcinoma with enteroblastic differentiation (GACED) lesion and the well-differentiated tubular adenocarcinoma lesion are adjacent to each other. A preoperative biopsy is performed with tissue samples from the site marked with ✖.

Contrast-enhanced computed tomography (CT) revealed thickening of the posterior wall of the gastric vestibule. Enlargement of lymph nodes at station 3, according to the Japanese Gastric Cancer Association (JGCA) guidelines, was observed. However, no distant metastases were detected. Based on these results, a cT3N1M0 cStage III (UICC TNM classification, 8th edition) of advanced gastric cancer of the lower stomach was diagnosed. Anemia due to tumor bleeding was noted, and surgical treatment was decided despite his advanced age. The patient underwent open distal gastrectomy (D1+ dissection, Roux-en-Y reconstruction). Intraperitoneal observation showed no liver metastases or disseminated metastases. No ascites was observed, and intraoperative peritoneal lavage cytology was performed. Invasion was suspected due to adhesions between the stomach and the transverse mesentery of the posterior wall of the stomach at the tumor site, and distal gastrectomy with combined resection of the anterior lobe of the transverse mesentery was performed. The reconstruction was performed using the Roux-en-Y technique. The operation time was 226 minutes, and the blood loss was 500 mL. On the 30th postoperative day, the patient was discharged home. In surgical specimens, type 3 lesions of 45×45 mm in the lower gastric region and 35 mm of type 0-IIa tumors (Paris classification) were found on the oral side of the type 3 lesions, but they were not detected preoperatively (Figure 2a).

**Figure 3:**
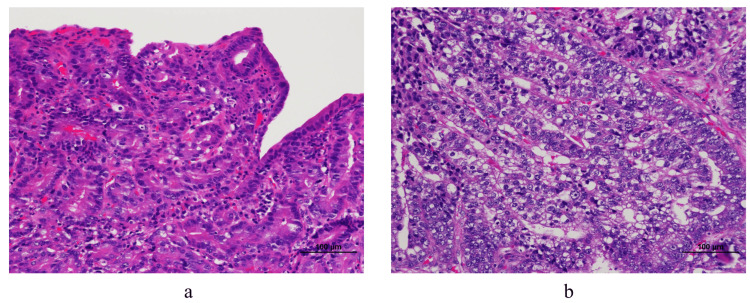
Histopathological findings (a) The type 0–IIa lesion showing a well-differentiated tubular adenocarcinoma (hematoxylin and eosin [H&E] stain ×200). (b) The type 3 lesion containing the columnar epithelium with a pale cytoplasm (H&E stain ×200).

Histopathological examination showed that the type 0-IIa lesion was well-differentiated tubular adenocarcinoma (Figure 3a), and the depth of the lesion was pT1a (UICC TNM classification, 8th edition). Type 3 lesions included the columnar epithelium with a pale cytoplasm (Figure 3b).

**Figure 4:**
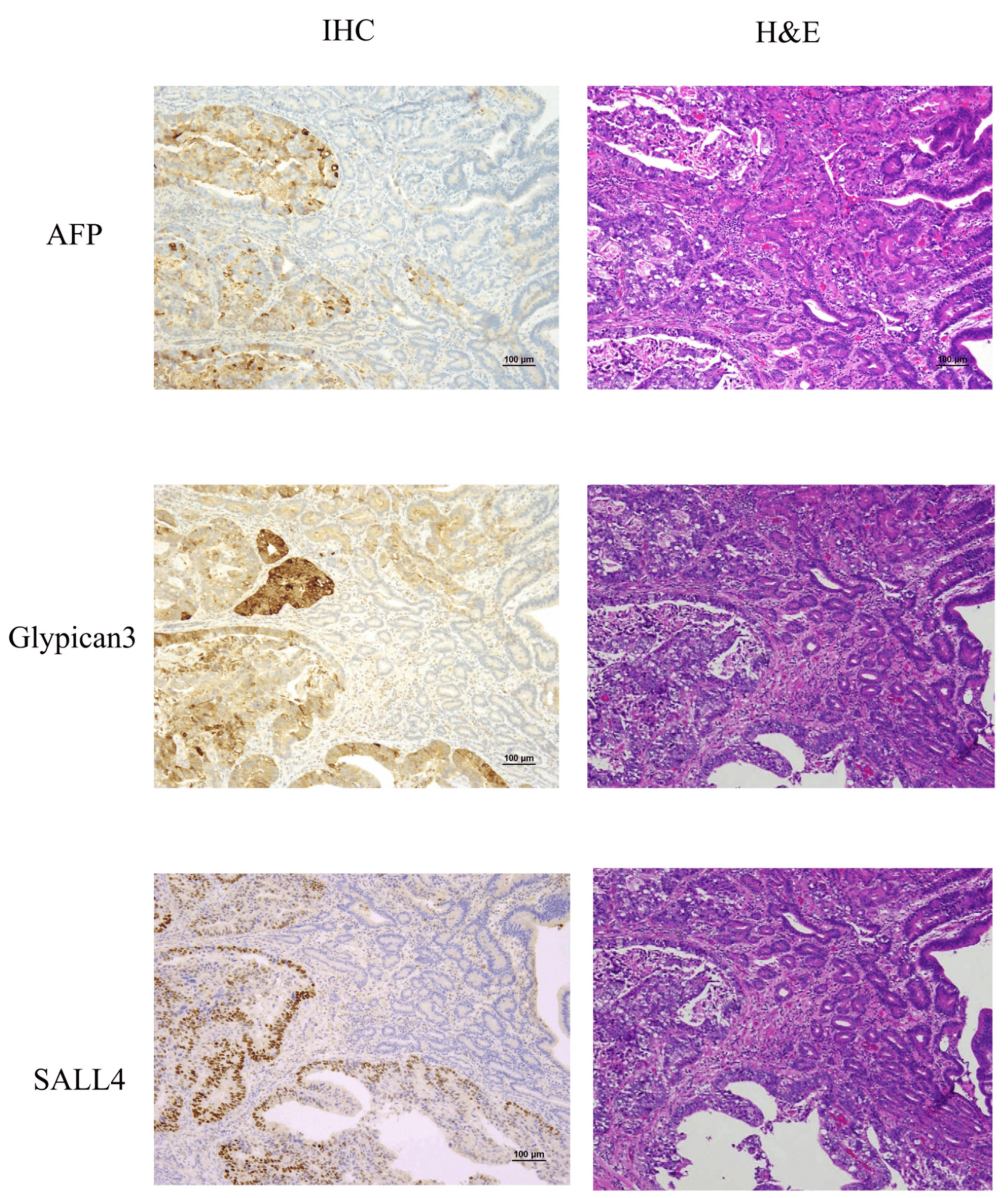
Immunohistological staining findings (GACED lesion) Immunostaining of the GACED lesions is partially positive for α-fetoprotein (AFP), Glypican-3, and SALL4. (AFP, Glypican-3, and SALL4; H&E stain ×100)

The depth of the lesion was pT3 (UICC TNM classification, 8th edition), and no evidence of transverse mesenteric invasion was noted. Type 3 lesions were partially positive for AFP, Glypican-3, and SALL4 by immunohistochemistry (IHC) and were diagnosed as GACED (Figure 4).

**Figure 5:**
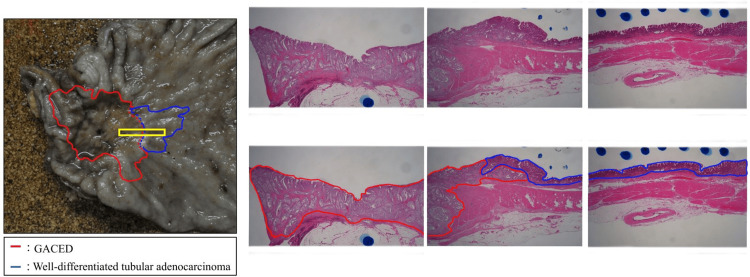
Histopathological findings at the border of both lesions At the border between the two lesions, tubular adenocarcinoma lesions are observed on the superficial layer, and GACED lesions are observed on the deeper layer.

The immunostainings were negative in the area of type 0-IIa lesions. No mixture of ductal adenocarcinoma components was observed in the area of the type 3 lesions. Sectioning and mapping of the specimen at the border of both lesions revealed that the ductal adenocarcinoma and GACED lesions were located adjacent to each other (Figure 2b). At the border between the two lesions, the ductal adenocarcinoma lesion was superficial, whereas the GACED lesion was located deep (Figure 5).

**Figure 6:**
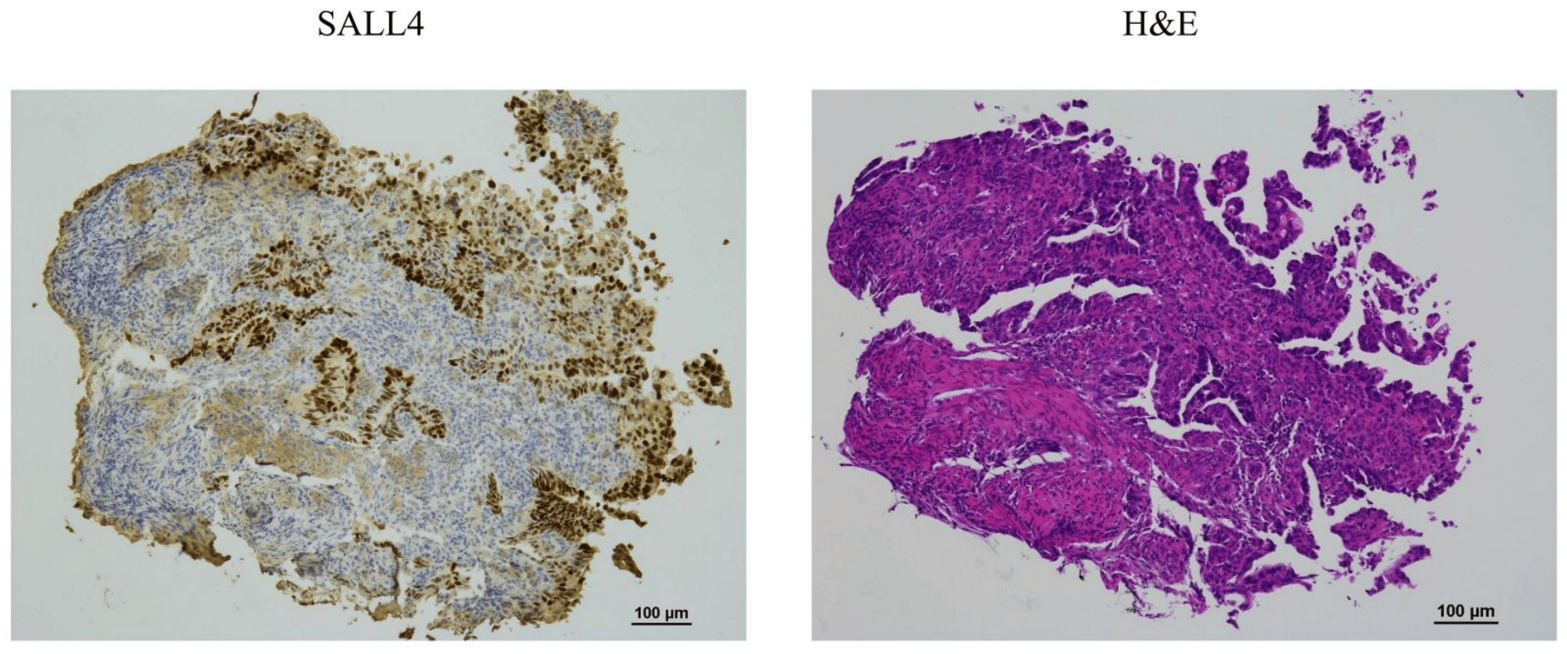
Immunohistological staining of the biopsy tissue The biopsy tissue is positive for SALL4 in most of the tumor gland ducts (SALL4, H&E stain ×100).

Metastases were found in 7 of the 26 dissected lymph nodes. And the diagnosis of pN3a (UICC TNM classification, 8th edition) was made. Lymph nodes at station 3 (according to the JGCA guidelines), which were enlarged on preoperative CT, were confirmed to be metastatic by histopathological examination. No lymphatic invasion was observed. However, venous infiltration was observed. Intraoperative peritoneal lavage cytology was positive. The patient was diagnosed with pStage IV (UICC TNM classification, 8th edition). Following the definitive diagnosis from the surgical specimen, additional immunohistochemical staining of the preoperative biopsy specimens revealed SALL4 positivity in the majority of tumor glandular ducts (Figure 6).

Postoperative chemotherapy was not indicated due to advanced age. At three months postoperatively, tumor markers were elevated (carcinoembryonic antigen, 40.7 ng/mL; CA19-9, 285.9 U/mL), and chest and abdominal CT showed lung and liver metastases. The patient did not request additional treatment, so no biopsy of metastases or other additional tumor marker tests were performed.​​​​​​​

## Discussion

The Japanese Gastric Cancer Treatment Regulations (15th edition) have classified GACED as a special type of malignant epithelial tumor. GACED is an adenocarcinoma with tubular, papillary, or full-blown growth of the columnar epithelium with a pale cytoplasm resembling a fetal gastrointestinal epithelium. Clinically, GACED is categorized as an AFP-producing gastric carcinoma, which also encompasses hepatic adenocarcinoma (HAC) and yolk sac tumor-like carcinoma. AFP-producing gastric cancer, a relatively rare malignant tumor type that accounts for 2.7%-5.4% of gastric cancers and has a high liver metastasis rate, is a high-grade cancer with a poor prognosis [4]. Murakami et al. [5], who identified 29 patients with GACED, defined GACED as a tubular, papillary, or full-grown adenocarcinoma that comprised cells resembling the fetal gastrointestinal epithelium with pale cytoplasm and positive for AFP, Glypican-3, or SALL4, which are expressed on the fetal gastrointestinal epithelium. They reported that GACED is a high-grade cancer that is associated with severe lymphatic (76%) and venous (72%) invasions as well as a high incidence of lymph node (69%) and liver (31%) metastases. Furthermore, Akazawa et al. [6] investigated 51 patients with GACED and reported a high incidence of lymphatic invasion (66.7%), venous invasion (68.7%), lymph node metastasis (70.6%), and liver metastasis (33.3%), reconfirming GACED as a high-grade cancer.

Using IHC, GACED is positive for AFP, Glypican-3, or SALL4, which are employed as diagnostic markers. AFP and Glypican-3 are highly expressed in fetal stem cells, and their expression is also noted in the intestinal epithelium in the early embryonic stages [7]. SALL4, which plays a significant role in maintaining embryonic stem cell pluripotency, is expressed in the intestinal epithelium in the early embryonic period (9-12 weeks) and gradually decreases and disappears thereafter [7]. Reportedly, the positivity rates of these markers in GACED are 45%, 83%, and 72% for AFP, Glypican-3, and SALL4, respectively [5].

In our case, tubular adenocarcinoma and GACED were observed adjacent to each other, with tubular adenocarcinoma on the superficial layer and GACED on the deeper layer at the border between the two lesions. When two cancer types are detected in proximity, as in the present case, collision or metaplasia from one cancer type to another represents the possible mechanism of their development and progression. AFP-producing gastric carcinomas, including GACED, are frequently associated with conventional adenocarcinomas, including papillary and tubular adenocarcinoma. Kinjo et al. [3] investigated 23 patients with AFP-producing gastric cancer and revealed that conventional adenocarcinoma was observed in the mucosa in more than 90% of the cases. Conversely, in the deeper layers, GACED and HAC were present in several cases, and conventional adenocarcinoma was absent in approximately 40% of the cases. Furthermore, the deeper invasive areas more frequently expressed AFP than the superficial areas, indicating that conventional adenocarcinomas may initially develop in the mucosa, acquire fetal-type traits, invade deeper layers, and subsequently transform into GACED and HAC [3,8].

In 1980, Spagnolo et al. [9] established the following diagnostic criteria for collisions: (1) the distribution of two distinct tissue types can be clearly distinguished, (2) each tissue type can be identified at adjacent sites, and (3) both components may be mixed at the collision site, suggesting that an area appearing as a transition of both components exists. In our case, tubular adenocarcinoma and GACED were observed on the superficial and deeper layers, respectively, at the boundary between the two lesions. Additionally, IHC staining was performed on each lesion, but no intermingling of the two components was identified. These findings are consistent with the possibility of a colliding carcinoma composed of conventional tubular adenocarcinoma and GACED.

The medical search for “gastric adenocarcinoma with enteroblastic differentiation” from 2000 to 2024 revealed 12 case reports [10-18]. The 13 cases, including the present case, are summarized in Table 1. Six of the seven available cases had coexisting conventional adenocarcinoma, suggesting a high coexistence rate of conventional adenocarcinoma in GACED, as previously reported. However, Yamada et al. [11] reported a case wherein conventional adenocarcinoma did not coexist and emphasized the possibility of de novo occurrence of GACED. Among the reports of GACED and conventional adenocarcinoma coexistence, our case is the only one wherein the two lesions were adjacent to each other, and there was no mixture of the two tumors except for the border area. In our case, GACED occurred de novo and collided with a well-differentiated tubular adenocarcinoma. In addition, eight of the 13 cases were pT1, among which lymphatic invasion was observed in four cases and distant metastasis in three cases. Consistent with previous reports, GACED was considered a high-grade tumor. Even in pT1 cases of GACED, careful follow-up with consideration of recurrence is warranted. Although biopsies were performed in seven cases, only two cases were diagnosed as GACED, suggesting that the preoperative diagnosis of GACED is difficult. Biopsy samples were obtained from the ulcer margin, rather than from the ulcer site of the type 3 lesion, suggesting that the GACED component is poorly exposed in the superficial dysplastic glandular ducts at the biopsy site. In our case, hematoxylin and eosin (H&E) staining of the biopsy tissue revealed no evidence of GACED, and additional IHC at a later date showed SALL4 positivity in the majority of the tumor gland ducts. Based on this, if GACED had been suspected preoperatively and additional immunohistochemical staining of the biopsy specimen had been performed, a preoperative diagnosis might have been possible. Recognition of this disease is considered essential for the preoperative diagnosis of GACED. Regarding preoperative diagnosis, Li et al. [19] analyzed 724 patients who underwent gastric cancer resection and reported that among 25 patients with a preoperative serum AFP level >10 μg/L, 11 (44%) were diagnosed with GACED. This suggests that preoperative measurement of serum AFP may serve as a useful indicator for the preoperative diagnosis of GACED.

**Table 1:** Reports of “gastric adenocarcinoma with enteroblastic differentiation" ESD: Endoscopic submucosal dissection/CY: Intraoperative peritoneal lavage cytology

Author	Year	Age	Sex	Macroscopic type	Size (mm)	Diagnosis of biopsy	Treatment	Coexistence of common adenocarcinoma type	Depth (pT)	pN	Ly	V	Metastasis
Yamabuki T [10]	2014	75	F	Type 2	20x15	tub2	Operation	N/A	T2	N1	N/A	N/A	Lymph node
Yamada R [11]	2018	73	F	Type 0-Ⅱa+Ⅱc	10x8	N/A	ESD followed by operation	-	T1b	N0	N/A	N/A	N/A
Iwaya M [12]	2020	72	M	Plypoid solid lesion	15x15	N/A	Operation	+	T1a	N/A	+	N/A	Liver(susp)
Iwaya M [12]	2020	77	M	Depressed lesion	16x8	N/A	Operation	+	T1b	N/A	N/A	N/A	Liver
Iwaya M [12]	2020	66	F	Slightly depressed lesion	15x10	N/A	Operation	+	T1b	N/A	N/A	N/A	None
Iwata H [13]	2023	77	M	Type 0-Ⅱc	15	N/A	ESD	N/A	T1b	N/A	-	-	None
Ngan RK [14]	2023	61	M	N/A	34	GACED	Chemotherapy	N/A	N/A	N/A	N/A	N/A	Lymph node, Liver, Adrenal gland
Ishikawa A [15]	2024	70	M	Superficial depressed lesion	20	N/A	ESD	N/A	T1b	N/A	+	N/A	None
Nakayama H [16]	2024	39	F	Type 3	88x32	Adenocarcinoma	Operation	N/A	T3	N0	+	-	None
Suzuki N [17]	2024	74	F	Type 2	N/A	GACED	Chemotherapy	N/A	N/A	N/A	N/A	N/A	Lung
Matsuyama S [18]	2024	70	M	Type 0-Ⅰ+Ⅱc	15	tub2	Operation	+	T1b	N0	+	-	None
Matsuyama S [18]	2024	70	M	Flat depressed lesion	10	tub2	ESD followed by operation	+	T1b	N2	+	+	Lymph node
Our case		92	M	Type 3	45x45	tub1	Operation	+	T3	N3a	-	+	Lymph node,CY, Liver,Lung

Data on the oncologic dynamics and prognosis of GACED remain lacking, and the same treatment as that for general-type adenocarcinoma has been employed. Nakayama et al. [16] reported a case of GACED with positive peritoneal washing cytology who survived for >5 years without recurrence after receiving postoperative chemotherapy, as in the case of general-type adenocarcinoma. Ngan [14] reported a case of de novo stage IV human epidermal growth factor 2 (HER2)-positive GACED in which good tumor control was achieved using anti-HER2 drugs.

In GACED, 34.6% of the patients demonstrated HER2 amplification by immunohistological staining and fluorescence in situ hybridization, and the relatively high HER2 positivity rate suggests that anti-HER2 antibodies can be effective against GACED [20]. Therefore, considering the relatively high HER2 positivity rate, treatment with anti-HER2 antibody drugs is anticipated to be effective. In our case, the operation was performed because of the presence of tumor hemorrhage, and postoperative chemotherapy was not performed owing to the patient’s advanced age. GACED appears to respond similarly to conventional gastric adenocarcinoma and can be effectively managed with a combination of surgery and chemotherapy. Preoperative diagnosis of GACED may allow for selection of appropriate treatment strategies, including anti-HER2 therapies, particularly in patients with HER2-positive tumors. This approach might be beneficial in selected cases, although further accumulation of clinical evidence is needed.

## Conclusions

GACED is a rare but highly aggressive subtype of gastric cancer that may arise not only through transformation from conventional adenocarcinoma but also de novo, as suggested by our case. Although diagnosis is challenging, immunohistochemical markers such as AFP, Glypican-3, and SALL4 are helpful. In addition, preoperative measurement of serum AFP may be useful for diagnostic purposes. GACED can easily develop distant metastases even in early-stage cases, indicating the need for careful follow-up to monitor for recurrence. Although HER2 status was not assessed in our case, HER2 overexpression has been reported in some GACED cases, suggesting that targeted therapies may be beneficial in such instances. The pathogenesis and tumor biology of GACED remain largely unclear, and further accumulation of clinical cases is needed to better understand this disease.
